# Validation of the LITHUANIAN version of the 19-item audit of diabetes dependent quality of life (ADDQOL – LT) questionnaire in patients with diabetes

**DOI:** 10.1186/s12955-018-1033-5

**Published:** 2018-11-01

**Authors:** Žydrūnė Visockienė, Laura Narkauskaitė-Nedzinskienė, Roma Puronaitė, Aldona Mikaliūkštienė

**Affiliations:** 1 Faculty of Medicine, Vilnius University, Vilnius, Lithuania; 2 Vilnius University Hospital Santaros Klinikos, Vilnius, Lithuania

**Keywords:** Validity, Reliability, ADDQOL, Quality of life, Lithuania

## Abstract

**Background:**

Currently there is no diabetes-specific quality of life (QOL) instrument available in Lithuanian language. We aimed to develop a Lithuanian version of a widely-used individualised instrument - the Audit of Diabetes Dependent Quality of Life questionnaire (ADDQOL-19) and assess the validity and reliability in patients with type 1 and type 2 diabetes mellitus (DM).

**Methods:**

This study was conducted at the Primary Care and Endocrinology Outpatient Clinics in Vilnius. The ADDQOL was translated from the original English (UK) into Lithuanian using a standardized methodology of forward and back translation. After cognitive “debriefing” the validity and reliability of LT-ADDQOL questionnaire were assessed in a sample of 138 diabetes patients. *Cronbach’s alpha* coefficient*,* factor analysis, independent t tests and ANOVA were used.

**Results:**

There were 106 participants with type 2 and 32 with type 1 DM included in the study with a mean age of 55.5 years (± 14.5) and 56.2% women. The *Cronbach’s alpha* coefficient was 0.908 and most of items loading values onto one single factor were larger than 0.40 (varied from 0.41 to 0.77), indicating good internal consistency and reliability of instrument.

**Conclusions:**

We developed the Lithuanian version of ADDQOL-19 which is a valid and reliable instrument to measure impact of diabetes on QOL. It could be further used by clinicians and researchers for comprehensive assessment of QOL in adults with diabetes.

## Background

Diabetes is a chronic metabolic disease having a strong negative impact on many aspects of patients’ lives. Poorly controlled diabetes mellitus (DM) is associated with increased rate of vascular complications, impaired patient quality of life, less satisfaction with treatment, and greater health care expense per patient [1]. In addition to diabetes-related complications, change in life style, physical well-being, quantity and quality of social relationships, intensive treatment regimen (multiple insulin injections), episodes or fear of hypoglycemia may lead to reduced quality of life (QOL) [2, 3]. Although clinical treatment mostly focuses on medical outcomes, QOL is recognized as an important patient-reported health outcome in people with diabetes [4–6] and is an important part of holistic approach of patient care.

Diabetes–specific instruments assessing the impact of diabetes on specific aspects of life commonly affected by diabetes and determining relevance and importance for the individuals’ QOL provides a genuine measure of diabetes-specific QOL as well as a generic QOL overview [7, 8]. There is number of diabetes-specific QOL questionnaires originally developed for English speaking patients [9–13]. The Audit of Diabetes Dependent Quality of Life (ADDQOL) is one of the most widely used scales of diabetes-specific QOL in different populations and cultures [14–18]. The questionnaire was originally designed in 1994 to cover 13 broad aspects of life likely to be influenced by diabetes [14] and developed further in the next decade and now has 19 domain specific items. ADDQOL questionnaire is linguistically validated into more than 60 languages, which offers great potential for international comparative research. So far, the latest 19-item ADDQOL questionnaire has been validated and used in different countries and cultural environment [3, 15, 19–21].

Despite the availability of several generic health-related quality of life instruments in Lithuanian language, there is no single questionnaire validated to assess diabetes-specific QOL in Lithuanian speaking patients.

**The aim** of this study was to develop a Lithuanian version of ADDQOL-19 questionnaire and to assess the validity and reliability of the instrument in patients with type 1 and type 2 diabetes.

## Methods

### Study population

This study was conducted at the Primary Care and Endocrinology Outpatient Clinics of Vilnius University Hospital Santaros Klinikos (VUHSK) as a part of Clinical Audit evaluating the effectiveness of diabetes care within described settings. All patients attending the outpatient clinics for routine visits within a specified period of time were approached by doctors and asked to fill in the LT-ADDQOL questionnaire if they met inclusion criteria: age ≥ 18 years; ability to comprehend and speak Lithuanian language, physician diagnosed type 1 or type 2 diabetes. Patients with secondary diabetes, gestational diabetes or whose previous important medical information was missing were excluded from the study. A total number of 156 patients were recruited during October 2013 and February 2014 of which 138 patients met the inclusion criteria and thus were included into further analysis.

The Clinical Audit evaluating the effectiveness of diabetes care at outpatient clinics of VUHSK was approved by the hospital administration and local ethics committee. Verbal consent to fill in the LT-ADDQOL questionnaire was obtained from each patient. The final Lithuanian version of ADDQOL aimed to retain similar psychometric properties to the original questionnaire.

In addition to the ADDQOL responses, data on patients’ demographic characteristics, education, time since diabetes diagnosis, diabetes type, existing diabetic complications, prescribed medicines and concomitant diseases were collected.

### Instrument

The ADDQOL questionnaire consists of two overview and 19 specific items. The two overview items assess general quality of life (GQOL) and diabetes dependent quality of life (DDQOL). The first item (GQOL) shows how respondents feel about their present quality of life (scale from + 3 to − 3, where + 3 means “excellent” and -3 means “extremely bad”). The second item (DDQOL) asks the patient to evaluate what their quality of life would be if he/she did not have diabetes (scale from -3 to +1, where -3 means “very much better” and + 1 means “worse”).

Each of 19 domain-specific items consists of 2 parts. In part “a” the individual rates the impact of diabetes on applicable domains (scale from − 3 to + 1, where − 3 reflects maximum negative impact and + 1 reflects a positive impact). In part “b” the respondent rates the importance of each specific domain (scale from 3 to 0, where 3 means “very important” and 0 means “not at all important”). The impact rating is multiplied by the corresponding importance rating to provide a weighted impact score for each domain from − 9 (maximum negative impact) to + 3 (maximum positive impact). Weighted impact scores of each individual are summed and divided by the number of applicable domains, to give an overall Average Weighted Impact (AWI) score. Selected domains (working life, holiday, family life, close personal relationship, sex life) have a “not applicable” (N/A) option. N/A responses are excluded from the scoring for that individual in statistical analysis. If one or both parts of a domain response are missing, a weighted impact score is computed for that item. However, AWI was calculated when no more than 6 responses were missing. General structure of ADDQOL questionnaire is presented in Table 1.

**Table 1:** General structure of ADDQoL questionnaire

Category	Variables	Questions number
Overview items	Present quality of life	I
	Diabetes-dependent quality of life	II
Diabetes specific questions	Impact of diabetes on a particular life	a
	The importance of life domain	b
Life domains	Leisure activities	1
	Working life	2
	Local or long-distance journeys	3
	Holiday	4
	Physically do	5
	Family life	6
	Friendship and social life	7
	Close personal relationship	8
	Sex life	9
	Physical appearance	10
	Self-confidence	11
	Motivation	12
	People’s reaction	13
	Feelings about the future	14
	Financial situation	15
	Living conditions	16
	Dependence on others	17
	Freedom to eat	18
	Freedom to drink	19

### Linguistic validation

After obtaining the developers’ authorization, the ADDQOL was translated from the source English (UK) into Lithuanian version using a standardized methodology of forward and back translation. The translation process was divided into 4 main phases:

- Phase 1 is the main forward and back translation stage;
- Phase 2 is the piloting stage;
- Phase 3 is the final review stage;
- Phase 4 is the finalisation stage.

The forward translation (FT) was conducted independently by two Lithuanian translators, both fluent in English. After the FT, two other bilingual translators were recruited to back translate (BT) the ADDQOL into English independently. Translation guidelines were provided and the reconciliation process of FT and BT was closely guided, consulted and helped by the author of the questionnaire and co-ordinating researchers of their team. Following the final reconciliation, a BT report was compiled and send to the co-ordinator. Afterdiscussion with the co-ordinator, a preliminary ADDQOL was reconciled. This preliminary version of ADDQOL was reviewed by endocrinologist then send to the co-ordinating psychologist and discussed until the consensus was reached and ADDQOL became a subject to cognitive debriefing. Interviews of cognitive debriefing were conducted and reviewed by a doctor endocrinologist in a five patients (various age, sex, education and type of diabetes). After several rounds of reconciliation, the approval was obtained, and the final Lithuanian version of the ADDQOL was produced.

### Statistical analyses

Descriptive statistics was computed to summarize the sociodemographic and clinical characteristics. The evaluation of scale structure was undertaken using unforced explanatory factor analysis with Varimax rotation and forced one - factor explanatory factor analysis. The first two overview items were not included in the factor analysis. Standardized Cronbach’s alpha coefficient described internal consistency and was used for reliability analysis. Independent t test was used to test differences in means between two patient’s groups, ANOVA test - to test differences of more than two independent samples. *P*-values of less than 0.05 were considered to indicate statistical significance.

Statistical analyses were performed using SPSS Windows 20.0 programme. Results were presented as means ± standard deviation (SD) if not stated otherwise.

## Results

There were 138 patients, more than a half were women included in the study. The age of respondents ranged from 19 to 86, with the mean of 55.5 years (± 14.5). The average glycosylated hemoglobin (HbA1c) was 8.2% (± 1.9) (66.1 ± 2.7 mmol/mol) and the mean duration of diabetes was 10.8 years (± 8.5) in the whole group. More demographic and clinical characteristics of the sample are shown in Table 2.

**Table 2:** Description of the sample (*N* = 138) and comparison AWI scores in demographic and clinical characteristic groups

Characteristics	N (%)	AWI Mean (SD)	*P* value	Domains with statistically significant difference
Demographic				
Women	77 (56.2)	−3.19 (1.87)	0.588	Sex life
Men	61 (43.8)	−3.01 (1.85)		
Age, years				
18–40	20 (15.0)	− 3.27 (1.48)	0.004	Leisure activities, physically do, motivation, people’s reaction, financial situation, freedom to drink
41–60	58 (43.6)	− 3.57 (2.09)		
> 60	55 (41.4)	− 2.42 (1.48)		
Education				
High school not completed	5 (3.8)	−2.27 (1.22)	0.183	Holidays
High school completed	29 (22.3)	−3.61 (1.99)		
Professional education	38 (29.2)	−3.11 (2.01)		
College/university	58 (44.6)	−2.76 (1.65)		
Clinical				
DM Type 1	31 (22.6)	−3.08 (1.51)	0.913	Motivation
DM Type 2	106 (77.4)	−3.12 (1.95)		
Treatment				
Oral therapy	61 (44.9)	−2.72 (1.75)	0.077	Dependence on others
Insulin	47 (34.6)	−3.42 (1.75)		
Combination	28 (20.6)	−3.51 (2.10)		
HbA1C, % (mmol/mol)				
< 7 (53)	26 (26.3)	−2.54 (1.89)	0.097	Leisure activities
≥7 (53)	73 (73.7)	−3.24 (1.81)		
Complications				
Yes	84 (60.9)	−3.21 (1.74)	0.413	Physically do
No	54 (39.1)	−2.94 (2.01)		

The mean for the general QOL score was 0.32 (± 0.96) which is between “good” and “neither good nor bad”. The most popular answer for GQOL was “neither good nor bad” which was used by 58 (42.3%) responders. The mean for the DDQOL score was − 1.80 (± 0.96) which is between “much better” and “a little better”. Forty seven responders (34.3%) used “much better” to describe what their quality of life would be if they did not have diabetes and this was the most frequently chosen option. The distributions of responses for impact and importance ratings and individual weighted impact scores for each of 19 domains are presented in Table 3.

**Table 3:** The distributions of responses for impact and importance rating and weighted impact score

Domains	Number	Mean (SD)			
	N/A	Missing	Impact rating	Importance rating	Weighted impact score
Leisure activities		6	−1.47 (0.98)	1.86 (0.70)	−2.95 (2.47)
Working life	46	6	− 1.60 (1.03)	2.33 (0.71)	−3.90 (2.93)
Local or long-distance journeys		7	−1.62 (1.05)	1.78 (0.79)	−3.08 (2.55)
Holiday	26	11	−1.48 (1.02)	2.07 (0.74)	−3.29 (2.82)
Physically do		12	−1.78 (1.00)	2.20 (0.67)	−4.13 (2.92)
Family life	2	8	−1.29 (1.14)	2.55 (0.60)	−3.48 (3.30)
Friendship and social life		9	−1.25 (1.11)	2.04 (0.65)	−2.79 (2.88)
Close personal relationship	16	11	−1.12 (1.09)	2.35 (0.62)	−2.77 (3.02)
Sex life	43	11	−1.37 (1.10)	2.07 (0.77)	−2.82 (2.78)
Physical appearance		6	−1.41 (0.99)	2.00 (0.75)	−3.07 (2.63)
Self-confidence		6	−1.41 (1.06)	2.20 (0.75)	−3.34 (3.03)
Motivation		9	−1.28 (1.01)	2.03 (0.72)	−2.86 (2.68)
People’s reaction		10	−0.61 (0.87)	1.70 (0.80)	−1.30 (2.12)
Feelings about the future		7	−1.69 (1.10)	2.19 (0.80)	−4.14 (3.17)
Financial situation		7	−1.38 (1.05)	2.30 (0.63)	−3.35 (2.98)
Living conditions		4	−1.24 (1.06)	2.16 (0.70)	−2.90 (2.78)
Dependence on others		13	−1.15 (1.08)	2.03 (0.86)	−2.46 (2.70)
Freedom to eat		7	−1.89 (0.98)	1.95 (0.75)	−4.07 (2.91)
Freedom to drink		10	−1.52 (1.03)	1.67 (0.81)	−3.09 (2.86)

The greatest unweighted negative impact diabetes had was on “freedom to eat” - 1.89 (± 0.98) and the least negative impact on “people’s reaction” -0.62 (± 0.87). The highest level of importance was attributed for “family life” 2.55 (± 0.60) and the lowest was for “freedom to drink” 1.67 (± 0.81). The AWI of − 4.14 (± 3.17) and − 1.30 (± 2.12) showed “feelings about the future” and “people’s reaction” to be the most and the least impacted QOL domains respectively.

The mean average weighted impact (AWI) score was − 3.02 (± 1.86). The statistically significant difference in AWI scores was found between age groups, with the highest negative impact of diabetes on QOL in patients of 41–60 years and no difference in other demographic and clinical variable groups. Significant impact of diabetes on different QOL domains was observed in age, gender, education, type of diabetes, treatment and complications groups (Table 2).

For the scale structure and reliability analysis 95 cases out of 138 were included (after N/A and missing values were excluded). The estimated *Cronbach’s alpha* coefficient was 0.908. The *Cronbach’s alpha* with each of the 19 items deleted ranged from 0.897 if “family life” was deleted to 0.909 if “freedom to drink” was deleted as shown in Table 4. Unforced factor analysis with *Varimax* rotation, generated five factors with eigenvalues > 1 (Table 5). Seven domains were loaded on factor 1, five – on factor 2, five – on factor 3, five – on factor 4 and two – on factor 5 (with factor loadings > 0.40). A five-factor solution explained 67.6% of variance.

**Table 4:** The Cronbach’s alpha with each of the 19 items deleted for the Lithuanian ADDQoL

Domains	Corrected item-total correction	Squared multiple correlation	Cronbach’s alpha if item deleted Overall α = 0.908
Leisure activities	0.574	0.473	0.902
Working life	0.500	0.490	0.904
Local or long-distance journeys	0.473	0.434	0.904
Holiday	0.421	0.586	0.906
Physically do	0.596	0.566	0.901
Family life	0.722	0.683	0.897
Friendship and social life	0.695	0.631	0.898
Close personal relationship	0.606	0.569	0.901
Sex life	0.518	0.529	0.903
Physical appearance	0.541	0.584	0.902
Self-confidence	0.630	0.656	0.900
Motivation	0.630	0.592	0.900
People’s reaction	0.633	0.524	0.901
Feelings about the future	0.659	0.534	0.899
Financial situation	0.586	0.628	0.901
Living conditions	0.589	0.508	0.901
Dependence on others	0.505	0.467	0.903
Freedom to eat	0.380	0.522	0.907
Freedom to drink	0.313	0.528	0.909

**Table 5:** Unforced factor analysis with Varimax rotation

Domains	Factors				
	1	2	3	4	5
Leisure activities		0.506			
Working life		0.645		0.508	
Local or long-distance journeys		0.743			
Holiday		0.844			
Physically do		0.493	0.431		
Family life	0.544		0.537		
Friendship and social life	0.418		0.603		
Close personal relationship			0.753		
Sex life			0.842		
Physical appearance				0.771	
Self-confidence				0.815	
Motivation				0.536	
People’s reaction	0.435			0.473	
Feelings about the future	0.519				
Financial situation	0.786				
Living conditions	0.631				
Dependence on others	0.722				
Freedom to eat					0.862
Freedom to drink					0.898
Eigenvalues	3.047	2.741	2.698	2.546	1.804
Variance explained	16.035	14.425	14.202	13.399	9.497
Cumulative variance	16.035	30.460	44.662	58.061	67.557
Factor loadings range (> 0.40)	0.418–0.786	0.493–0.844	0.431–0.842	0.473–0.815	0.862–0.898
Factor loadings range (suppressed)	−0.145 - 0.376	−0.017 - 0.366	−0.012 - 0.368	−0.060 - 0.297	−0.042 - 0.302
Alpha	0.855	0.797	0.833	0.810	0.782

Kaiser-Meyer-Olkin measure of sampling adequacy: 0.849

Bartlett’s test of sphericity: χ2, d.f; *p*-value = 861.281, 171; *p* < 0.001

Eighteen items loaded onto one single factor using forced-one-factor analysis, with factor loadings > 0.40 (0.41 to 0.77). The item “freedom to drink” did not load highly on this factor (factor loading = 0.33), but if removed, *Cronbach’s alpha* increased only from 0.908 to 0.909. Thus all items were retained and scored in the single AWI score as for the original English ADDQOL19.

General QOL score was lower in patients with diabetic complications compared to those without complications (0.11 and 0.60 respectively, *p* < 0.01) and in elder age, compared to younger participants with the lowest score in > 60 years age group (0.65 in 18–40 years, 0.24 in 41–60 and 0.22 in > 60 years, *p* = 0.024). The statistically significant greater diabetes specific negative impact of diabetes on QOL was observed in patients with poor glycemic control (mean DDQOL score − 1.96 in HbA1c ≥7% vs − 1.46 in HbA1c < 7% group, p = 0.02) and those with high school completed (− 1.60 in high school not completed, − 2.28 in high school competed, − 1.58 in professional education and − 1.82 in college/university group, *p* = 0.29).

## Discussion

The aim of the study was to investigate the psychometric properties of the 19-item LT-ADDQOL questionnaire by determining the validity and reliability of the instrument among patients with DM in a primary care setting. Reliability in the current study was assessed to investigate internal consistency of the scale with an acceptable value of 0.908 obtained, which was comparable to original English version (0.85) [14]. Similar to other studies where standardized *Cronbach’s alpha* coefficient varies from 0.88 to 0.947 [3, 4, 8, 19–25] our results show that the instrument is reliable. Factor analysis also showed good results on multiple aspects of the ADDQOL scale, suggesting that all items were adequately linguistically validated. Pilot testing with clinicians and patients proved ADDQOL questionnaire to be valid for the Lithuanian population.

Generally, diabetes has a negative impact on quality of life. The results from our study revealed that people with diabetes in Lithuania had worse GQOL and diabetes had a greater negative impact on their QOL (DDQOL) compared to other countries: 0.3 and − 1.86 respectively vs. 1.10 and − 1.10 compared to Australia [3], 1.06 and − 1.31 compared to Norway [4] and 0.84 and − 1.26 compared to Great Britain [7].

Diabetes had a negative impact not only on patients’ overall Quality of Life (DDQOL item), but also on all other domains in our study. The greatest diabetes weighted negative impact was indicated for “feelings about the future” followed by “physically do”, “freedom to eat” and “working life”. Similar domains: “freedom to eat”, “feelings about the future” and “working life” had the lowest AWI scores in Norwegian version of the ADDQOL with the addition of the domain “freedom to drink” [4]; “freedom to eat”, “feelings about the future”, “working life” - in Slovak version with the addition of the domain “financial situation” [21]; “freedom to eat”, “feelings about the future”, “physically do” - in the Slovenian version with the addition of the domain “journeys” [25]. Interestingly, the greatest diabetes weighted negative impact in Chinese speaking populations was indicated for slightly different domains - “financial situation”, “family life”, “self-confidence” and “freedom to eat” in mainland China [19]; in the Taiwan population the most negatively impacted domains were “feelings about the future”, “family life”, “self-confidence” and “freedom to eat” [6]. These differences could be attributed to cultural differences and the funding of diabetes care.

In summary, the recurrent domain with low AWI score almost in all populations was “freedom to eat”, which indicates a strong influence of dietary restrictions on QOL, bearing in mind that being overweight is one of the most important factors contributing to the development of DMT2 [25]. The least negative AWI score in our study was estimated for “people’s reaction”, which was in line with already mentioned studies in the Slovenian and Slovak populations.

Although the greatest negative unweighted impact was found in the domain “freedom to eat” in our study and agreed with the results from Singapore [5], Portugal [8], Slovakia [21], Slovenia [25] and Greece [26], the highest importance score was attributed to “family life”. The same result of family having the highest importance rating was found in Singapore [5], China [19], Slovakia [21] and Greece [26], while Slovenian [25] participants scored “dependence on others” as the most important. Comparison of various ADDQOL domain scores between different studies showed quite significant variation in the means of impact, importance and weighted impact scores. This could be explained by different sociodemographic characteristics (ethnicity, education level, incomes), patients age (varied from 18 to 89 years with different proportions within each study population), diabetes duration, proportion of type 1 and type 2 diabetes subjects and proportion of patients with oral and insulin therapy. Senior participants and patients with diabetes complications reported worse general quality of life in our study, diabetes had significantly higher negative impact on more QOL domains in participants of > 61 years, compared to younger ones, differences in motivation were found in type 1 and type 2 diabetes participants.

Lower QOL in older diabetes participants found in the Lithuanian cohort is in line with other studies, where the ADDQOL questionnaire was used [26]. Also, the data from previous studies in Lithuania, where general QOL questionnaires were used, have shown lower rating in all fields of QOL by pensioners compared to blue-collar and white-collar workers [27]. However, two recent studies showed that younger age was associated with lower ADDQOL scores in Korean T2D patients and that being younger was associated with a greater negative impact of diabetes on QOL [28]. Maybe younger people are more afraid about their future, because their life is just beginning. The study in Greece showed that no statistically significant relations between QOL and sex, duration of diabetes, BMI, HbA1c, smoking habits, education level, antidiabetic treatment and diabetic complications [26]. On the other hand, the previous Lithuanian study showed that men and those with higher education evaluated all fields of quality of life better; age and body mass index are less important factors that can influence quality of life [27]. A possible explanation for this difference between men and women and those with more vs less education might be differences in economic status and access to health care systems.

Our experience in collecting the data shows that only a few participants reported difficulties in filling in the questionnaire. However, there were still some missing responses. The items “working life” and “sex life” in the ADDQOL had the most missing data. The missing response for “sex life” is understandable because most of the participants in the study are older and they do not usually discuss sexuality-related topics with their doctors, and younger participants may be shy to talk about it.

There is a need for further research in a bigger group of diabetes participants, to better understand the possible influencing factors, affecting diabetes-related quality of life and pointing out the most important psychological and clinical aspects that need to be addressed to improve patient’s quality of life.

## Conclusion

In conclusion, our results show that the Lithuanian version LT-ADDQOL has maintained its original psychometric properties and achieved adequate reliability and validity. Therefore, it could be recommended for use and further evaluation in quality of life research.
